# Macleayins A From *Macleaya* Promotes Cell Apoptosis Through Wnt/β-Catenin Signaling Pathway and Inhibits Proliferation, Migration, and Invasion in Cervical Cancer HeLa Cells

**DOI:** 10.3389/fphar.2021.668348

**Published:** 2021-08-06

**Authors:** Chunmei Sai, Wei Qin, Junyu Meng, Li-Na Gao, Lufen Huang, Zhen Zhang, Huannan Wang, Haixia Chen, Chaohua Yan

**Affiliations:** College of Pharmacy, Jining Medical University, Rizhao, China

**Keywords:** Macleayins A, *Macleaya*, anticervical cancer, Wnt/β-catenin cascade, proliferation, apoptosis, migration, invasion

## Abstract

Macleayins A (MA), a novel compound, was isolated from *Macleaya cordata* (Willd.) R. Br. *and Macleaya microcarpa* (Maxim.) Fedde. The plant species are the member of *Papaveraceae* family and have been used traditionally for diverse therapeutic purposes. According to the reported studies, the chemical constituents, as well as crude extracts of these plants, could attenuate the proliferation of several cancer cell lines, such as HL-60, A549, HepG2, and MCF-7. The current study aimed to investigate the anticervical cancer activity of MA and its related molecular mechanism. Isolation of MA was carried out using various column chromatographic methods, and its structure was elucidated with ^1^H NMR. The cytotoxicity of MA was determined against HeLa cell lines *via* CCK-8 assay. The cell proliferation, apoptosis, cell cycle, migration, and invasion were measured by EdU labeling, Annexin-V APC/7-AAD double staining, PI staining, and transwell assay, respectively. The protein expression levels of c-Myc, *β*-catenin, cyclin D1, and MMP-7 in the cells were evaluated by western blotting. The Wnt/β-catenin signaling cascade activation was verified using the Dual-Glo® Luciferase assay. We found that MA inhibited the growth of HeLa cells at 72 h (IC_50_ = 26.88 µM) *via* inducing apoptotic process, reduced the proliferation rate by 29.89%, and decreased the cells migration and invasion as compared to the untreated group. It arrested the cell cycle at the G1 phase and its treatment inhibited the expression of related proteins c-Myc, *β*-catenin, cyclin D1, and MMP-7 in the Wnt/β-catenin signaling cascade. Further, the Wnt/β-catenin signaling cascade activation in MA-treated HeLa cells was attenuated in a dose-dependent manner. These findings demonstrate the anticancer effects of MA on a mechanistic level, thus providing a basis for MA to become a potential candidate drug for resistance of cervical carcinoma.

**GRAPHICAL ABSTRACT:**
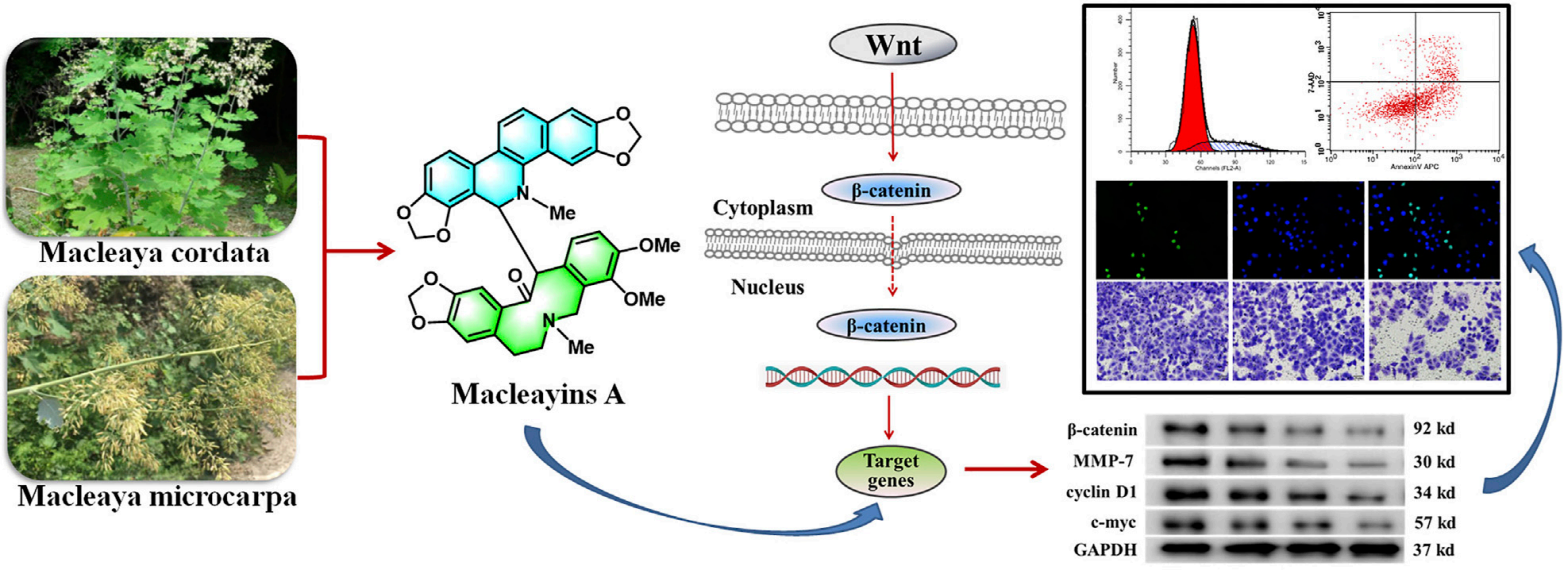


## Introduction

According to the report of Lancet Global Health 2019, about 570,000 cervical cancer cases and 311,000 cervical cancer deaths occurred in 2018 across the globe, with 106,000 cases and 48,000 deaths reported in China. Despite vaccination campaigns against human papillomavirus (HPV), cervical carcinoma has been considered to be one of the third most prevalent carcinomas in the world. Mostly, cervical carcinoma is found to be affecting women below the age of 45 years (Siegel et al., 2019; Arbyn et al., 2020; de Felice et al., 2021). According to the stage of disease at diagnosis, the treatment strategies of cervical carcinoma particularly comprised of surgery, chemotherapy, immunotherapy, radiotherapy, and locally targeted therapy (Liu et al., 2020a; de Felice et al., 2021). However, limited treatment options can be proposed in the case of recurrent or metastatic disease. Therefore, the research on invasion and metastasis of cervical cancer has been a hot topic in recent years. The patients are often treated with chemotherapy with or without bevacizumab, but survival time is still shorter (17 months) and associated with a rapid deterioration of quality of life due to toxicity profile. (Marchetti, et al., 2018; Marchetti et al., 2020). Therefore, researchers are currently focusing on investigating the candidate drugs with relatively low toxicity for overcoming cervical carcinoma. Epidemiology and animal experiments have shown that various natural ingredients can block the occurrence and development of tumors. Hence, it is not only of theoretical value but also of practical significance to search for antitumor drugs from plant active ingredients having high efficiency and low toxicity (Bich Ngoc et al., 2020).

*Macleaya cordata* (Willd.) R. Br. and *M. microcarpa* (Maxim.) Fedde. plant species are the members of the *Papaveraceae* family. Among *Papaveraceae*, the most commonly known and extensively studied species is *Macleaya cordata*. It is a perennial herb and is widely found in Japan, Northwest, and South China, whereas the origin of *M. microcarpa* is in central China (Chinese Academy of Sciences, Chinese Flora Chronicle Committee, 1998; Lin et al., 2020). As traditional medical herbs in China, the majority of studies have focused on *Macleaya* due to structurally diverse and biologically active alkaloids, and their various medicinal applications, such as antitumor, anti-inflammatory, antimicrobial, and insecticidal (Lin et al., 2017; Liu Y. et al., 2020; Nguyen et al., 2020). In China, *M. cordata* has also been used to treat cancer, including thyroid cancer and cervical cancer (Ke et al., 2017; Jiang et al., 2020).

Our previous results on the chemical constituents of *M. cordata* suggested that the new compound MA suppressed cell growth of many human cancer cells, including leukemia, lung, and liver cancer (Sai et al., 2015). The compound was again isolated as part of our ongoing research on the chemical constituents of *M. microcarpa*. The antitumor activity and mechanism of this compound were further studied and the obtained results revealed that MA inhibited the growth of the HeLa cell line in a concentration-dependent manner with an IC_50_ value of 26.88 μM. Previous reports have shown that the ingredients of *M. cordata* can induce and stabilize G-quadruplex made in the proximal promoter site of the *MYC* oncogene, thereby inhibiting the expression of MYC (Gao et al., 2020). The protein expressed by MYC works as a transcription factor and is associated with cell proliferation, metastasis, and apoptotic process. This transcription factor considerably contributes to the metastasis of tumors (Wang K.-B. et al., 2019). C-Myc was one of the key downstream target proteins in the Wnt signaling cascade (Ji et al., 2013). It has been revealed that Wnt/β-catenin signaling cascade activation in an abnormal manner triggers the initiation and progression of the tumor (Wang et al., 2018). Herein, we aim to explore the underlying mechanisms through which MA regulates the Wnt/β-catenin signaling cascade, causing decreased proliferation, invasion, metastasis, and induced apoptosis of cervical carcinoma cell lines.

## Materials and Methods

### Plant Material

The *M. microcarpa* fruits were obtained from Xiaguan Town, Neixiang, Nanyang County (located in the southwest of Henan province, China), following the national and institutional rules concerning biodiversity rights. The above plant sample was identified and the voucher sample (XGBLH-20170918) was preserved in the pharmaceutical experimental center, Jining Medical University, Rizhao, China.

### Preparation of Phytochemical Constituent

The fruits of *M. microcarpa* were air-dried, followed by grinding into a coarse powder. The powder (15.0 Kg) was soaked (three times) in 95% ethanol (18 L) (Guanglian, China) for 7 days at around 20–22°C. The extracts were combined and filtered, followed by concentrating using a rotary evaporator (Yiheng, China). The obtained concentrated ethanolic extract was suspended in water, followed by extracting with petroleum ether (PE) (Fuyu, China), methylene chloride (CH_2_Cl_2_) (Yuandong, China), and n-BuOH (Fuyu, China). Then these diluted extracts were concentrated under vacuum to obtain PE, CH_2_Cl_2_, and *n*-BuOH extracts. DCM extract (365 g) was fractioned *via* silica gel column chromatography (CC) and eluted with PE (60–90°C)–ethyl acetate (100:5, 100:10, 100:20, 100:50, 1:1 and 0:100, v/v) to get six fractions (Fr.A-Fr.F). Fr.E was further separated by MCI gel (Mitsubishi, Japan) CC eluting with MeOH–H_2_O (70:30, 80:20, 90:10, 100:0) to afford four subfractions (E1–E4). Subfraction E3 was purified using Sephadex LH-20 with CH_2_Cl_2_: MeOH (1:1) to yield compound 1 (40 mg).

The ^1^H NMR spectra of compound 1 were measured on Bruker ARX-300 NMR spectrometers (Bruker, Switzerland). The TLC and NMR data of compound 1 were the same as MA, which was previously isolated from *M. cordata*. The structural identification of the new compound, i.e., MA, has been published in Organic Letters in 2015 by our team (Sai et al., 2015).

### Cell Lines and Culture Medium

Cell lines, i.e., HeLa, SiHa (human cervical cancer), HepG2 (human liver cancer), and HFL-1 (human normal cells-human embryonic lung fibroblast) were procured from Jiangsu KeyGEN BioTECH Co., Ltd. (Nanjing, China). The HeLa, SiHa, and HepG2 cell lines were grown in 90% MEM medium, containing 10 percent of FBS. HFL-1 cells were cultured in 90% F-12K medium, containing 10% FBS. The cells incubation was carried out in humidified atmosphere (5% CO_2_) at 37°C.

### *In Vitro* Cytotoxicity Assay and Determination of IC_50_

The cytotoxicity effect of MA was examined *via* CCK-8 assay (Wang X. et al., 2019). Cells were digested, counted, and prepared into cell suspension of 5.0 ×10^4^ cells/mL. After that, 5.0 ×10^5^ cells were seeded into 96-well microtiter plates, followed by incubating for 24 h in the presence of 5 percent CO_2_ and 37°C temperature. The drug MA was diluted to different concentrations (100, 50, 25, 12.5, 6.25, 3.125, 1.56, 0.78, and 0.39 μM) with the complete medium; then, 100 μL of each MA dilution were added into each well. The negative control group was established. After 72 h of cultivation, following transfection, experimental wells were incubated with 10 μL/well of CCK-8 for 3 h. The absorbance was measured at a wavelength of 450 nm *via* a microplate reader (BioTek ELx800, United States). The following formula was employed to calculate the percent inhibition.

Inhibition rate (%) = (OD Negative control group - OD Experimental group)/OD Negative control group×100%

The experiments were independently repeated three times. The cytotoxic ability of MA was assessed with IC_50_ obtained from probability unit weighted regression method using SPSS (Staffstical Package for the Social Science) 24.0. The results have been indicated as the mean ± SD.

### Cell Proliferation Assay by EdU Labeling

The proliferation experiment on the HeLa cell line was performed *via* EdU labeling using keyFluor488Click-iT EdU Staining Proliferation Kit (KGA330, Jiangsu KeyGEN BioTECH Co., Ltd., China), following the provided instructions of the manufacturer (Li et al., 2019; Shao et al., 2020). Cell solution (1×10^4^/ml, 200 μL) was seeded into a 96-well plate (Corning Incorporated 3599, United States). After 24 h of incubation (in the presence of 5 percent CO_2_ and 37°C temperature), different concentrations (i.e., 0, 6.75, 13.5, and 27.0 μM) of MA-containing medium were added and cultured for 72 h. Next, the removal of media was carried out, followed by washing of cells *via* PBS twice. The addition of EDU solution (50 μM prepared with the medium) was carried out into each well, and then the incubation of cells was performed at 37°C and CO_2_ (5%) for 2 h. The medium was discarded, followed by rewashing (twice) with PBS. Cell fixatives (i.e., PBS containing 4% paraformaldehyde, 50 μL) were added to each well and incubated for 0.5 h at approximately 20–22°C. Next, the fixative solution was discarded. Glycine (50 μL, 2 mg/ml) was added into each well and treated for 5 min in a decolorizing cradle (WH-2, Shanghai Huxi Analytical Instrument Factory, China) and then discarded. PBS (100 μL) was added to each well, washed with a decolorizing cradle for 5 min, and then discarded. 1 × Apollo® staining reaction solution (100 μL) was added to each well and incubated with a decolorizing cradle for 0.5 h at approximately 20–22°C in dark, and discarded. Penetrant (0.5% TritonX-100 PBS, 100 μL) was added, washed by decolorizing cradle for 2–3 times (10 min each time), and then discarded. 1 × Hoechst 33,342 reaction cocktail (100 μL) was added to each well and incubated in a decolorizing cradle for 30 min in dark at room temperature and then discarded. Images were visualized under a high-content cell imaging system (200×) (MD, United States) and combined *via* Adobe Photoshop, version 6.0. Furthermore, EdU-positive cells, as well as total cells, were calculated in each chamber. All experiments were repeated three times.

### Cell Apoptosis Assay by Annexin-V APC/7-AAD Double Staining Method

Cell apoptosis was detected by Annexin-V APC/7-AAD double staining assay (Kong et al., 2019; Lin et al., 2019). HeLa cells in the logarithmic growth phase were digested and inoculated into six-well cell culture plates (Corning Incorporated 3516, United States). The next day, after cells attached to the bottom of the plate, the underlined cells were treated with the corresponding drug-containing medium (0, 6.75, 13.5, 27.0 μM) at 37°C for 72 h. The cells were digested with 0.25% trypsin (without EDTA) and then washed twice with PBS (centrifugation at 1,000 rpm for 5 min), and 5×10^5^ cells were collected. The cells were then resuspended in 500 µL of binding buffer. Finally, Annexin V–APC/7-AAD (KGA1024, Jiangsu KeyGEN BioTECH Co., Ltd., China) was added and mixed well at around 20–22°C for 15 min in the absence of light. The apoptotic process of the cells was evaluated *via* flow cytometry (Becton-Dickinson FACS Calibur, United States). All experiments were repeated thricely.

### Cell Cycle Assay by a PI Staining Method

The cell cycle distribution in the presence of MA was measured by propidium iodide (PI) staining method using flow cytometry (Zhang et al., 2018). HeLa cells (5.0×10^4^ cells/mL) were plated in 6-well plates, followed by 24 h incubation. The cells were then exposed to various concentrations (i.e., 0, 6.75, 13.5, and 27.0 μM) of MA at 37°C for 72 h. The cells were washed with PBS buffer and then collected and fixed with 70% EtOH for 24 h at −4°C. The fixed cells were rewashed *via* PBS, followed by 100 μL RNase-A exposure at 37°C for hrs., and, at the end, the cells were stained *via* 400 μL PI in the absence of light for 30 min at 4°C. The red fluorescence at the excitation wavelength of 488 nm was recorded by a flow cytometer (Becton-Dickinson FACS Calibur, United States). All experiments were repeated thricely.

### Cell Migration and Invasion Assays

Cell migration and invasion abilities were evaluated using transwell chamber (Corning Incorporated 3422, United States) [in the absence of Matrigel for transwell migration assay/with Matrigel (BD 356234, United States) for transwell invasion assay] (Ji et al., 2013). The cells of logarithmic growth phase were digested and inoculated into a six-well plate. The next day, after the cells adhered to the wall, the serum was removed and the cells were starved in incomplete medium for 24 h. Place Matrigel adhesive at 4°C to melt overnight. Dilute the melted Matrigel glue twice with incomplete medium, add 30 μL diluted Matrigel in the upper chamber of transwell, and incubate at 37°C for 120 min to polymerize Matrigel into glue. Cells were digested and counted, and the cell density was adjusted to 1×10^5^ cells/mL. The above was the Matrigel laminating process in cell invasion assay. The following were the same experimental procedures for cell migration and invasion. The following are the same experimental procedures for invasion and migration experiments. Cell suspension (100 μL) was added into the above chamber, and at the same time, 500 μL medium (comprised of FBS) was supplemented to each well of 24-well plate (Corning Incorporated 3514, United States) in the lower chamber. The plates were incubated for 24 h, followed by eliminating the upper membrane of cells *via* a cotton swab, and then transwell was removed, inverted, and air-dried, accordingly. Those migrated/invaded cells in the 24-well plates were fixed and stained with 500 μL of 0.1 percent crystal violet (Sigma C3886, United States), the chamber was plated in the dye, and the membrane was immersed in the dye for 30 min at 37°C and washed with PBS. The images of each well (containing cells) were taken and quantified in three random fields using an inverted biological microscope (at ×200 magnification, IX51, Olympus, Japan). Experiments were performed three times.

### The Analysis of Protein Expression by Western Blotting

Western blotting was performed for analyzing protein expression (Zhang et al., 2013; Ma et al., 2018). The proteins were extracted by using the Whole Cell Lysis Assay KGP 250 (Jiangsu KeyGEN BioTECH Co., Ltd.), as suggested by the manufacturer. HeLa cells were harvested following treatment with macleayin A for 72 h. The proteins were extracted in lysis buffer. Protein quantification was evaluated *via* BCA Protein Assay Kit (KGA902, Jiangsu KeyGEN BioTECH Co., Ltd.). An SDS-PAGE was carried out for the separation of proteins, followed by electroblotting onto the NC membrane. Next, the membrane blockage was carried out by skimmed milk (5%) for 2 h. Membranes were incubated at 4°C for 24 h with the underlined primary antibodies: rabbit anti-β-catenin (1:5,000; Abcam; cat. no. ab32572), rabbit anti-c-Myc (1:1,000; Abcam; cat. no. ab32072), rabbit anti-cyclin D1 (1:10,000; Abcam; cat. no. ab134175), rabbit anti-MMP-7 (1:1,000; Abcam; cat. no. ab207299), and rabbit anti-GAPDH (1:5,000; cat. no. KGAA002; Jiangsu KeyGEN BioTECH Co., Ltd., China). GAPDH served as an internal control. Next, the membranes incubation was carried out with secondary antibodies, i.e., Goat Anti-Rabbit lgG HRP (KGAA35) at 25°C for 2 h. Enhanced chemiluminescence (ECL) detection system was employed for visualizing immunoreactivity. Imaging was carried out using Syngene G: Box Chemixr5 (Britain), and the grayscale of the results was examined *via* Gel-Pro32 software. All experiments were performed in triplicate.

### Luciferase Assay

The culturing of HeLa cells (5×10^4^ cells/well) was carried out in the presence of MA in 12-well plates. After overnight incubation, the underlined cells were cotransfected with Topflash firefly luciferase plasmid. Cells were transfected *via* Lipofectamine™ 3000 reagent. Next, the transfection of the cells was carried out for 24 h. After 72 h of MA treatment, cells were subjected to a luciferase-kit- (i.e., Dual-Glo® Luciferase assay kit, E2920, Promega) based assay according to the provided procedure of the manufacturer. The normalized firefly luciferase assay was evaluated as the quotient of firefly/Renilla luciferase activity. Experiments were performed in triplicate.

## Results

### Analysis of Phytochemical Constituent Macleayins A

**FIGURE 1:**
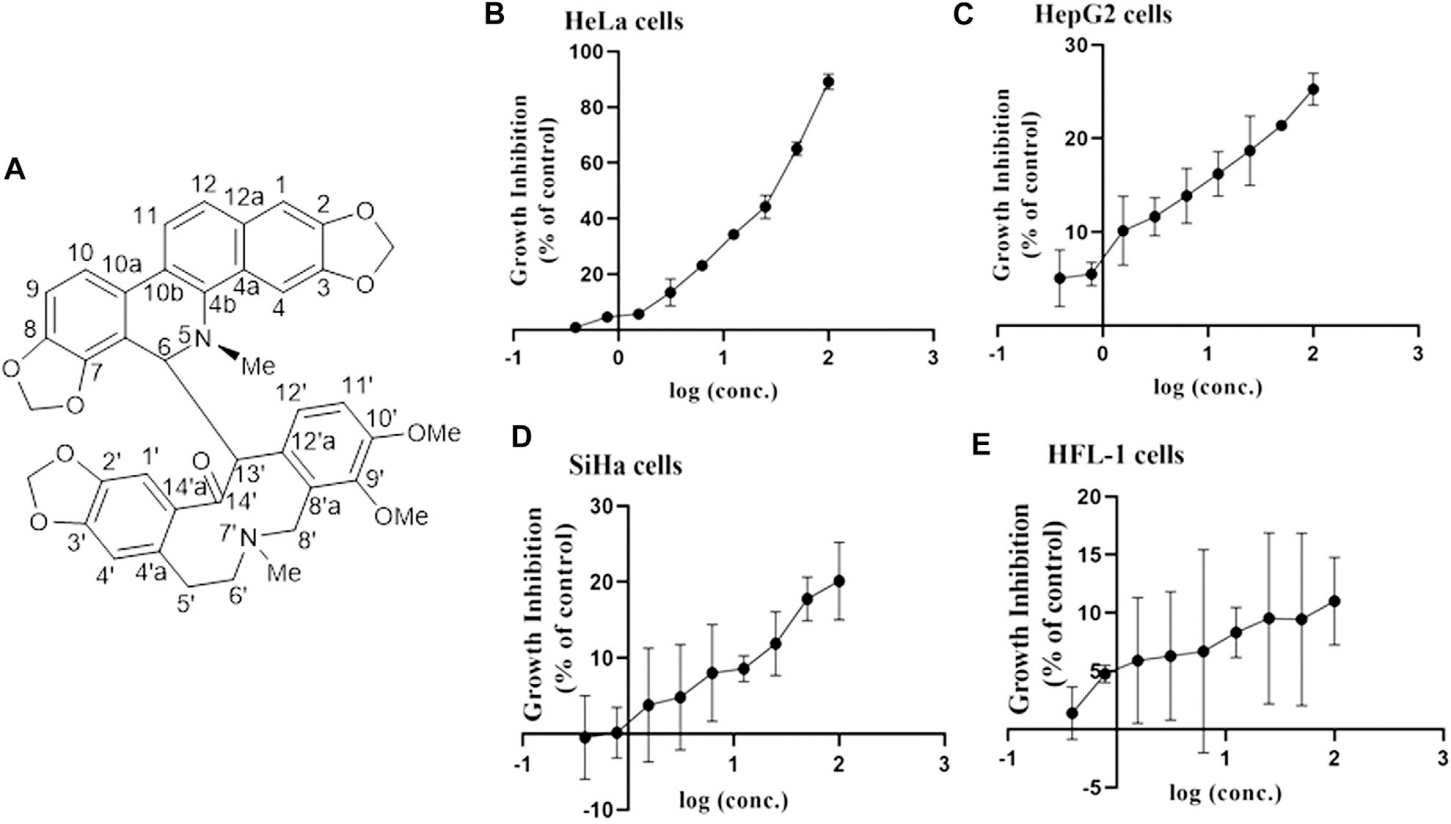
Structure of MA and the association between inhibition of growth and MA concentrations on various cell lines. **(A)** Structure of MA. **(B)** HeLa cervical carcinoma cell line. **(C)** HepG2 liver carcinoma cell line. **(D)** SiHa cervical carcinoma cell line. **(E)** HFL-1 normal cell line. Each dot indicates the mean ± SD of triplicate experiments that were evaluated independently.

MA: This compound was obtained as a white powder with a molecular formula, i.e., C_41_H_36_N_2_O_9_. The underlined compound was observed as a dark speck under UV light with a wavelength of 254 nm by using silica gel TLC plates and Dragendorff’s reagent. ^1^H NMR (400 MHz, CDCl_3_) *δ*: 7.01 (1H, s, H-1), 6.61 (1H, s, H-4), 4.81 (1H, d, J = 11.0 Hz, H-6), 6.57 (1H, br d, J = 7.7 Hz, H-9), 7.12 (1H, d, J = 8.1 Hz, H-10), 7.68 (1H, d, J = 8.6 Hz, H-11), 7.46 (1H, d, J = 8.6 Hz, H-12), 6.23 (1H, s, H-4′), 2.57 (1H, brs, H-5′a), 1.96 (1H, d, J = 16.3 Hz, H-5′b), 1.83 (1H, brs, H-6′a), 2.39 (1H, d, J = 12.6 Hz, H-6′b), 2.32 (1H, brs, H-8′a), 3.07 (1H, d, J = 13.4 Hz, H-8′b), 7.07 (1H, d, J = 8.5 Hz, H-11′), 7.52 (1H, brs, H-12′), 4.53 (1H, brs, H-13′), 2.49 (3H, s, 5-N-CH3), 1.52 (3H, s, 7′-N-CH3), 5.91 (1H, d, J = 1.1 Hz, 2,3-OCH2O-), 5.88 (1H, d, J = 1.1 Hz, 2,3-OCH2O-), 6.11 (1H, d, J = 1.5 Hz, 7,8-OCH2O-), 5.95 (1H, d, J = 1.3 Hz, 7,8-OCH2O-), 5.92 (1H, d, J = 1.5 Hz, 2′,3′-OCH2O-), 5.90 (1H, d, J = 1.5 Hz, 2′,3′-OCH2O-), 3.46 (3H, s, 9′-OCH3), 3.95 (3H, s, 10′-OCH3). The structure was shown in Figure 1A.

### Cytotoxicity of Macleayins A

To investigate the antitumor activity of MA obtained from *M. microcarpa*, we first evaluated the cytotoxicity of candidate compound, i.e., MA, on different cancer cell lines. CCK-8 assay was employed to evaluate the effects of MA on the feasibility of HeLa, SiHa, HepG2, and HFL-1cells by exposing cells to various concentrations of MA, i.e., 0.39–100 µM for 72 h, respectively. The obtained results indicated that the viability of HeLa cells was considerably decreased than Siha and HepG2 cells in a dose-dependent manner, and MA had little effect on the viability of normal cell HFL-1, as depicted in Figures 1B–E. The IC_50_ value of MA against HeLa cells was 26.88 µM. Therefore, HeLa cells were selected for further studies on cancer and its associated mechanisms.

### Effect of Macleayins A on Proliferation, Apoptosis, Migration, and Invasion of HeLa Cells

**FIGURE 2:**
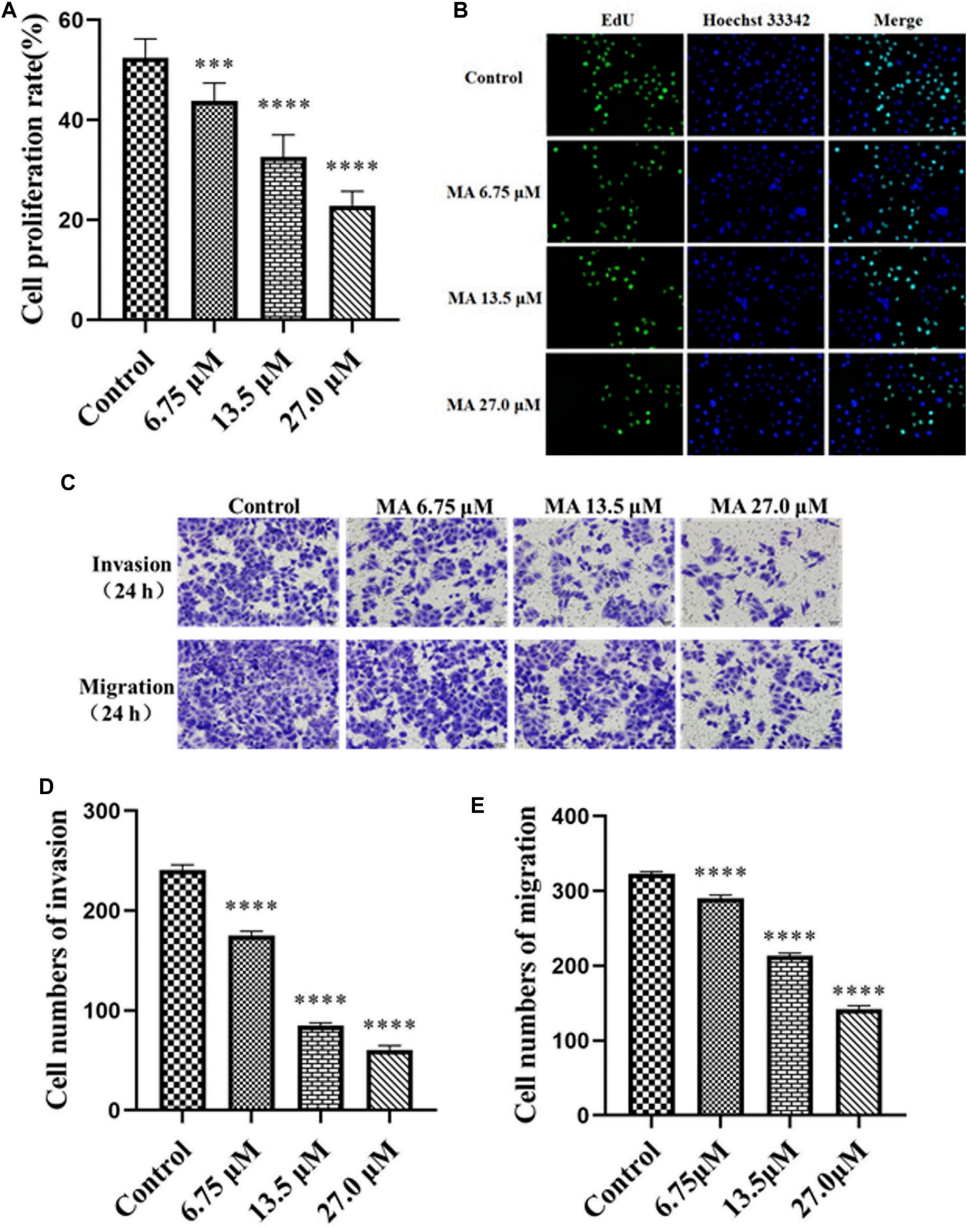
MA inhibits the proliferation, invasion, and migration of HeLa cells. **(A)** The proliferation rate of cells treated with MA. **(B)** Images by EdU assay: cells DNA (blue) stained with Hoechst 33,342 (ab145597), green color revealed EdU/Hoechst-positive cells. **(C)** Images of cell invasion and migration by transwell assay. **(D)** The quantitative analysis of the cell invasion. **(E)** The quantitative analysis of the cell migration. Control represents the blank control group without any treatment. The results obtained from triplicate experiments (independent) are represented as mean ± SD (magnification ×200; relative to the control group, ***p < 0.001, **** *p* < 0.0001).

To further investigate the effects of MA on cervical carcinoma, various concentrations of MA were used and our results indicated an IC_50_ value of 27.0 µM against the HeLa cells. We have selected the various concentrations of MA, i.e., 6.75, 13.5, and 27.0 µM, for evaluating the effect of MA on the proliferation, apoptosis, invasion, and migration of the carcinoma cells. Furthermore, we examined whether the reduced number of HeLa cells by MA (in CCK-8 assays) was caused by inhibiting proliferation or inducing apoptosis. To detect and quantify the effect of MA on the attenuation of cells proliferation, EdU assay was used and the cell densities (treated with MA for 72 h) were monitored through high content cell imaging system and it has been revealed that MA inhibited EdU incorporated cell proportion in a concentration-dependent manner, as depicted in Figures 2A,B.

**FIGURE 3:**
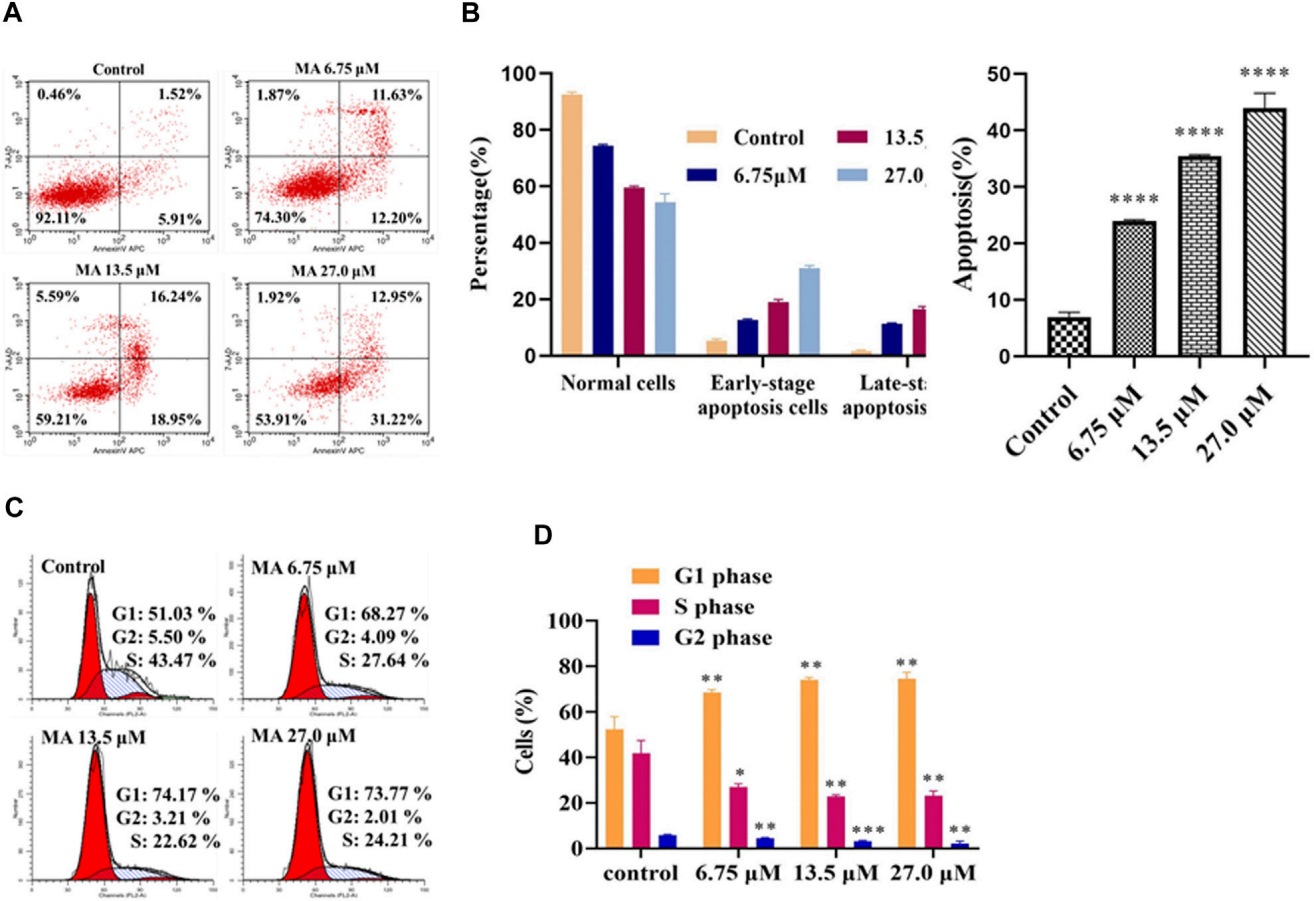
MA enhanced the apoptotic process of HeLa cells and stimulated cell cycle arrest of HeLa cells in G0/G1 phase. **(A)** Apoptotic profile of cells treated with different concentrations of MA by Flow cytometer (Annexin-V APC/7-AAD). **Upper left** quadrant, naked nucleus cell; **upper right** quadrant, late apoptosis or necrotic cells; **lower left** quadrant, living cells; **lower right** quadrant, early apoptotic cell. **(B)** The quantitative analysis of the proportion of apoptosis was carried out. **(C)** Cell cycle distribution (left red, right red, oblique line, and white part indicate G1 phase cell, G2 phase cells, S phase cells, and total cells, accordingly). **(D)** The quantitative analysis of different phases has been indicated. The results obtained from triplicate experiments (independent) were represented as mean ± SD (relative to the control group, *p < 0.05, **p < 0.01, ***p < 0.001, ****p < 0.0001).

Activation of apoptotic cell death is considered an effective approach for the treatment of cancer cells (Bich Ngoc et al., 2020). The results of apoptosis assay (by Annexin-V APC/7-AAD double staining) revealed that the elevation in the concentration of MA considerably elevated the proportion of apoptotic cells (early as well as late apoptotic cells), i.e., 7.43, 23.83, 35.19, and 44.17% for 0, 6.75, 13.5, and 27 μM, accordingly, as depicted in Figures 3A,B. The underlined results suggested that MA induced apoptosis in HeLa cells. The above results also indicated that MA can suppress the proliferation of HeLa cells by activating the apoptotic process.

Tumor migration and invasion are necessary stages in tumorigenesis and are the most life-threatening features of cancer. The cervical cancer cells become metastatic, resulting in the migration and invasion to other tissues (Liang et al., 2016; Zhang et al., 2018). Next, we performed a transwell assay to investigate the effects of MA on HeLa cells migration and invasion. The obtained results revealed that the invasion and migration abilities of cells reduced post-MA exposure relative to the negative control group in a concentration-dependent manner, as depicted in Figures 2C–E. These results suggested that MA can inhibit invasion and migration in HeLa cells.

### Macleayins A Stimulates G0/G1 Cell Cycle Arrest in HeLa Cells

The obtained results revealed that MA could stimulate the apoptotic process in HeLa cells. Next, we performed flow cytometry (*via* PI staining) to examine cell cycle arrest and to explore the inhibitory mechanism of cell growth. As shown in Figure 3C, the proportion of HeLa cells (not treated with MA) in the G1 phase was 51.03%. However, when these cells were exposed to MA, a considerable elevation was observed in the percentage of the underlined cells in a concentration-dependent manner, i.e., 68.27, 74.17, and 73.77% with 6.75, 13.5, and 27.0 μM, accordingly, as depicted in Figure 3D, while, in S-phase, a considerable downward trend was observed in these cells, which revealed that MA can block the cell cycle in the G1 phase.

### Macleayins A Inhibits the Activation of Wnt/β-Catenin Signaling in Cervical Carcinoma

**FIGURE 4:**
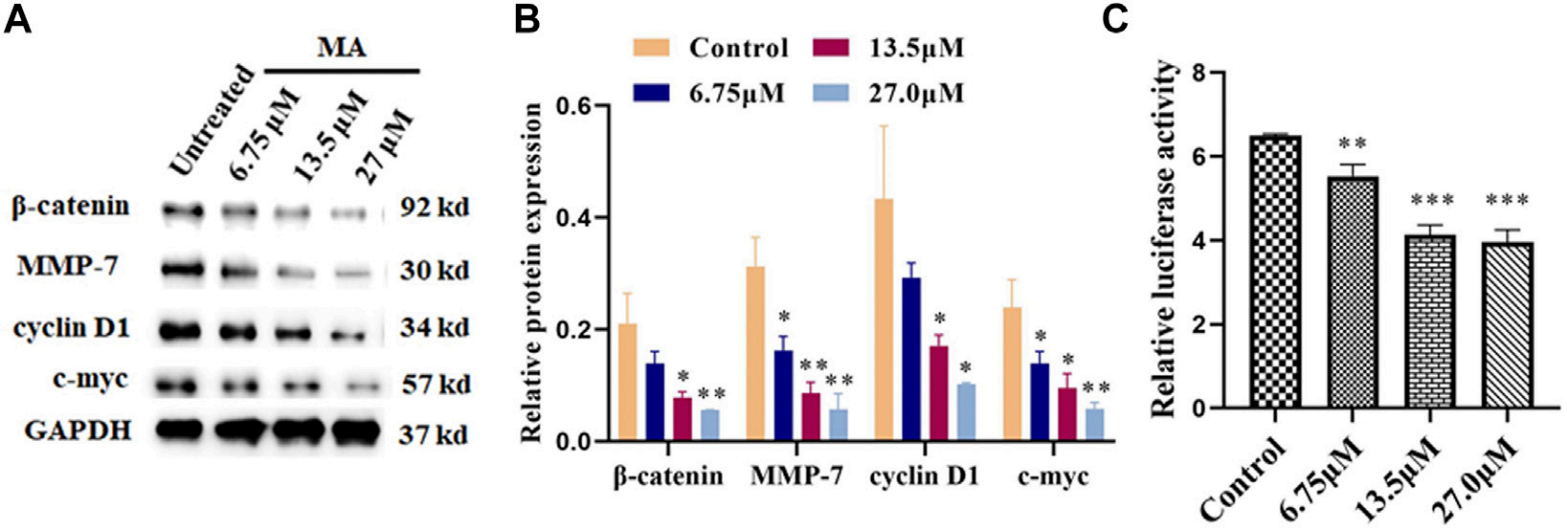
MA inhibits the activation of Wnt/*β*-catenin signaling. **(A)** Western blotting images: MA decreases the protein expression levels of *β*-catenin, cyclin D1, MMP-7, and c-Myc. GAPDH was used as internal control. **(B)** Quantification of associated proteins. **(C)** MA decreases the luciferase activity of the Topflash firefly luciferase. The obtained results were expressed as means ± SD (n = 3) (relative to the control group, *p < 0.05, **p < 0.01, ***p < 0.001).

To further explore the effects of MA on the molecular mechanisms regarding the HeLa cells proliferation, apoptosis, migration, and invasion, we examined the expression of associated proteins. Western blot results showed that the protein expression levels of *β*-catenin, cyclin D1, MMP-7, and c-Myc prominently decreased after MA treatment in a dose-dependent manner (Figures 4A,B) which suggested the contribution of MA against the proliferation of cervical cancer cells through downregulating the expression level of the critical protein, i.e., *β*-catenin, cyclin D1, MMP-7, and c-Myc of Wnt/β-catenin signaling cascade.

Topflash reporter plasmid has been used to examine the level of *β*-catenin-mediated TCF/LEF transcription activity in the Wnt signaling cascade. Topflash is widely used to study Wnt signaling pathway (Liang et al., 2016; Xu et al., 2016). To further elucidate the direct effect of MA on Wnt/β-catenin signaling, luciferase activity was examined after cotransfection of Topflash luciferase plasmids for 12 h and HeLa cells treatment with MA for 72 h using a Dual-Glo Luciferase Assay System. As shown in Figure 4C, MA considerably decreased the luciferase activity of the Topflash firefly luciferase.

## Discussion

Cervical cancer is the fourth highly prevalent malignancy among women, and it has been an alarming disease due to its high rate of recurrence after surgery. (Liu M.-M. et al., 2020). Currently, the main treatments for cervical cancer include chemotherapy, immunotherapy, and surgical resection, while other approaches include traditional Chinese medicine (TCM). Many TCMs are having many medicinal functions that cause improvement in symptoms of cancer and decrease cancer metastasis and the risk of cancer recurrence. The effective natural products obtained from TCM have been revealed as an effective candidate against tumors (Yu et al., 2019; Yang et al., 2020). In addition, many natural products have been developed into antitumor drugs, such as paclitaxel, camptothecin, and vincristine. However, there is a need to explore their therapeutic targets and associated molecular mechanisms. Previous reports on *Macleaya* treatment of cervical cancer paid much attention to their alkaloids with anticancer activity (Jiang et al., 2020). MA is a new compound extracted from *M. cordata* and *M. microcarpa*. In our previous study, it has been revealed that MA could inhibit the growth of HL-60, A-549, MCF-7, and HeLa tumor cells, which indicated that it has potential anticancer effects. In this study, it has been revealed that MA has antitumor effects by regulating the Wnt/β-catenin signaling cascade; attenuating the proliferation, invasion, and metastasis of cervical cancer cell lines; and inducing apoptosis, which can provide a theoretical basis for evaluating potential new natural antitumor products.

In the current study, the results obtained from CCK-8 and EdU assay revealed that elevated concentrations of MA can attenuate the cancer cell proliferation through activating the apoptotic process of the HeLa cell, and the average ratio of the underlined cells apoptosis was elevated to 44%. The underlined effect was further validated by Annexin-V APC/7-AAD double staining. Early and late apoptotic cells were evaluated through flow cytometry by using Annexin V-labeled fluorescent dye APC and 7-AAD, accordingly. Therefore, the apoptosis rate in our experimental results refers to the sum of early and late apoptosis, namely the sum of apoptosis rates shown on the upper right and the lower right quadrant. It has been known that the molecular mechanism of apoptosis has been categorized into endogenous death receptor cascade and exogenous mitochondrial cascade, the core part of the apoptotic process in mitochondria. Many of their features make it possible to be used as targets to kill cancer cells (Wu et al. 2019). They are not only a sensor of the endogenous apoptotic pathway, but also act as an amplifier of apoptotic signals, allowing cell apoptosis to proceed quickly and efficiently. Up to date, the natural active products have been associated with apoptosis that exerts their antitumor effects by activating apoptosis and attenuating proliferation, invasion, and metastasis in target cells as well as tissues (Yang et al., 2017). C-Myc is an oncogene with malignant transformation, and c-Myc protein (transcription factor) has been considerably contributed to the differentiation, growth, and apoptotic process of the cell. C-Myc proto-oncogene amplification is closely related to tumor formation, development, and metastasis and is highly expressed in cervical cancer, breast cancer, gastric cancer, and other tumors (Souza et al., 2013). The main characteristic of the mitochondrial apoptotic cascade is the dysregulation of MMP. The reported studies have revealed that the MMP-7 expression has been closely associated with tumor invasion and metastasis (Sharma et al., 2012; Liu Y. et al., 2020). In the current study, the obtained results of the transwell assay revealed that the migration, as well as invasion of HeLa cells, was effectively attenuated (malignant and invasive cell numbers decreased by 180 relative to the untreated group). Under MA treatment, we further verified that the protein expression levels of c-Myc and MMP-7 were inhibited with the increased concentration of MA (in a concentration-dependent manner) by western blot assay.

Based on the changes in cell DNA content, the growth and reproduction of proliferating cells can be divided into four stages: G1, S, G2, and M (mitosis) phase; these phases are collectively called the cell cycle (Niyonizigiye et al., 2019; Zhang et al., 2019). The proliferating cells in the tumor undergo changes in the cell cycle. Currently, researchers are trying to explore various therapeutic measures for tumor cell proliferation at different stages of the cell cycle (Sharma et al., 2012). The uncontrolled cell cycle is one of the obvious features of tumor cells, which was evaluated by identifying the inhibitory effect of MA treatment on the cell cycle *via* PI staining through flow cytometry. The obtained results revealed that the cell cycle of HeLa cells (exposed to MA) was arrested in the G1 phase (cells elevated by 22.15% than the group not exposed to MA). Cyclin D1 is a cell cycle machine and growth factor sensor that considerably contributes to the development of the G1 to S phase (Liu M.-M. et al., 2020). We analyzed the expression of a protein associated with the cell cycle arrest by western blotting and found that the expression of Cyclin D1 was reduced.

The Wnt signaling pathway is a complex pathway that regulates cell growth and proliferation (Xu, et al., 2016). The abnormal excitation of the pathway due to genetic mutation or increased stability can activate the abnormal expression of downstream target genes, including Cyclin, C-Myc, and MMP-7, which can lead to cell proliferation, inhibition of cell apoptosis, and tumor formation (Ji et al., 2013). Canonical Wnt/β-catenin pathway activates gene transcription through *β*-catenin (Zhang et al., 2013). Nuclear *β*-catenin turning on genes that promote cell division is a component of adherens junctions that interact with E-cadherin. *β*-Catenin (in free form) regulates the expression of genes by entering the nucleus. Abnormal expression or activation of *β*-catenin can cause a tumor. An elevated expression of *β*-catenin has been reported to increase the migration and invasiveness of tumor cells. Wnt signaling pathway is one of the signal transduction pathways that participate in the mechanism of tumorigenesis and has a key contribution in signal transduction, cell cycle, growth, migration, and apoptotic process, etc. (Yang et al., 2017). In this study, MA has been shown as a potential candidate against cervical cancer by suppressing the expression level of MMP-7, *β*-catenin, c-Myc, and cyclin D1 in Wnt signaling cascade. Topflash reporter plasmid has been used to determine the *β*-catenin-mediated TCF/LEF transcription activity in the Wnt signaling cascade and has been widely used to study Wnt signaling cascade (Xu et al., 2016). Therefore, we used the Topflash firefly luciferase plasmid (Sigma) through Dual Glo® luciferase reporter gene assay to further verify whether MA plays an anticervical cancer role by inhibiting the stimulation of Wnt/β-catenin cascade. The experimental results showed that MA significantly suppressed the luciferase activity of the Topflash firefly luciferase, which implied that MA can inhibit the triggering of the Wnt/β-catenin cascade. In short, our findings suggested that MA (from *Macleaya*) is a candidate natural product against cervical cancer that may delay the progression of cervical cancer through the Wnt/β-catenin signaling cascade; restrain the proliferation, migration, and invasion; and enhance the apoptotic process of cells.

In conclusion, MA, a bioactive compound of plant origin, can considerably attenuate the growth and development of cervical cancer cells, induce apoptosis, and cause cell cycle arrest in the G0/G1 phase which indicates its higher antitumor efficacy. Furthermore, the obtained results also revealed that the migration and invasion of cells were attenuated by MA exposure. Regarding the mechanism, MA might attenuate the proliferation of cells by downregulating the expression level of MMP-7, *β*-catenin, cyclin D1, and c-Myc and considerably attenuating apoptotic process in cervical cancer cells through Wnt/β-catenin signaling cascade. Thus, MA from *Macleaya* has been suggested as a potential candidate against cervical cancer.

## Data Availability

The original contributions presented in the study are included in the article/Supplementary Material; further inquiries can be directed to the corresponding author.
