# Supplementary material for: Macleayins A From Macleaya Promotes Cell Apoptosis Through Wnt/β-Catenin Signaling Pathway and Inhibits Proliferation, Migration, and Invasion in Cervical Cancer HeLa Cells
Source: Front Pharmacol. 2021 Aug 6;12:668348. doi: 10.3389/fphar.2021.668348 (PMC8377739; doi:10.3389/fphar.2021.668348)
Supplement: Supplementary file 1 [file DataSheet1.PDF]

frontiers

Supplementary Material

Macleayins A from *Macleaya* promotes cell apoptosis through Wnt/β-catenin signaling pathway and inhibits proliferation, migration, invasion in cervical cancer HeLa cells

Chunmei Sai[1,*], Wei Qin[1], Junyu Meng[1], Lina Gao[1], Lufen Huang[1], Zhen Zhang[1], Huannan Wang[1],Haixia Chen[1], Chaohua Yan[1]

[1] College of Pharmacy, Jining Medical University, Rizhao, Shandong, China

Corresponding Author

Chunmei Sai

saichunmei1980@163.com (Chunmei Sai).

Contents

1 The ^{1}H NMR spectra of Macleayins A 3
2 Data of in vitro cytotoxicity assay and determination by CCK-8 3
3 Data of cell proliferation assay by EdU labeling method.. 7
4 Data of cell apoptosis assay by Annexin-V APC/7-AAD double staining method ... 9
5 Data of cell cycle assay by PI staining method 9
6 Data of cell migration and invasion assay by transwell 9
7 Data of protein expression assay by Western blot... 10
8 Data of Luciferase reporter assay 11

Supplementary Figures and Tables

1 The ^{1}H NMR spectra of Macleayins A

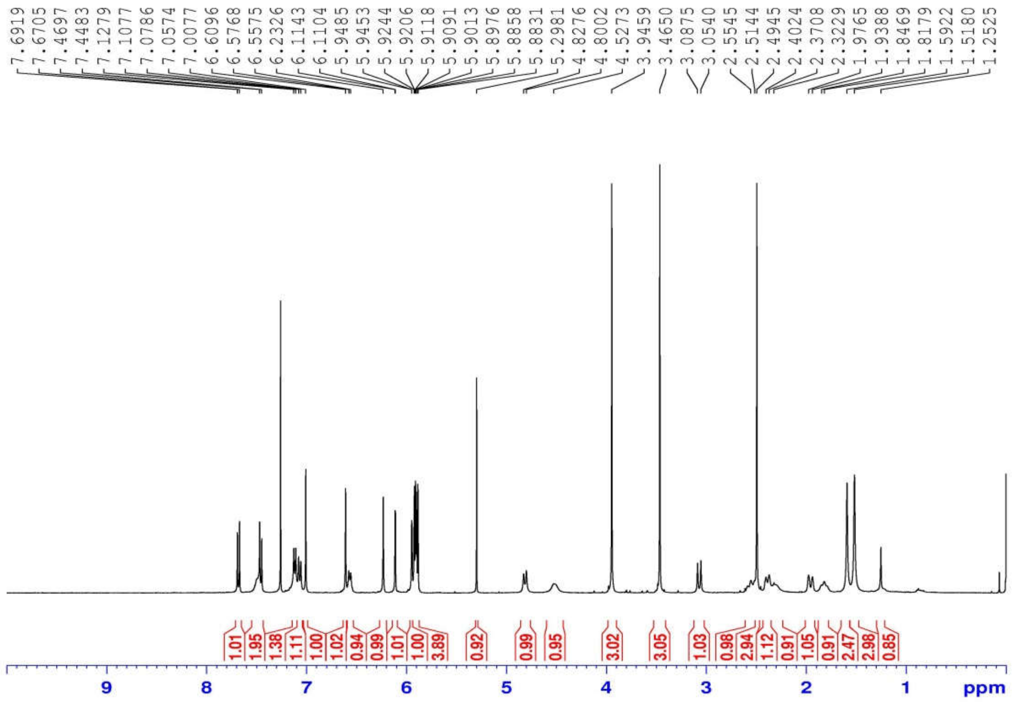

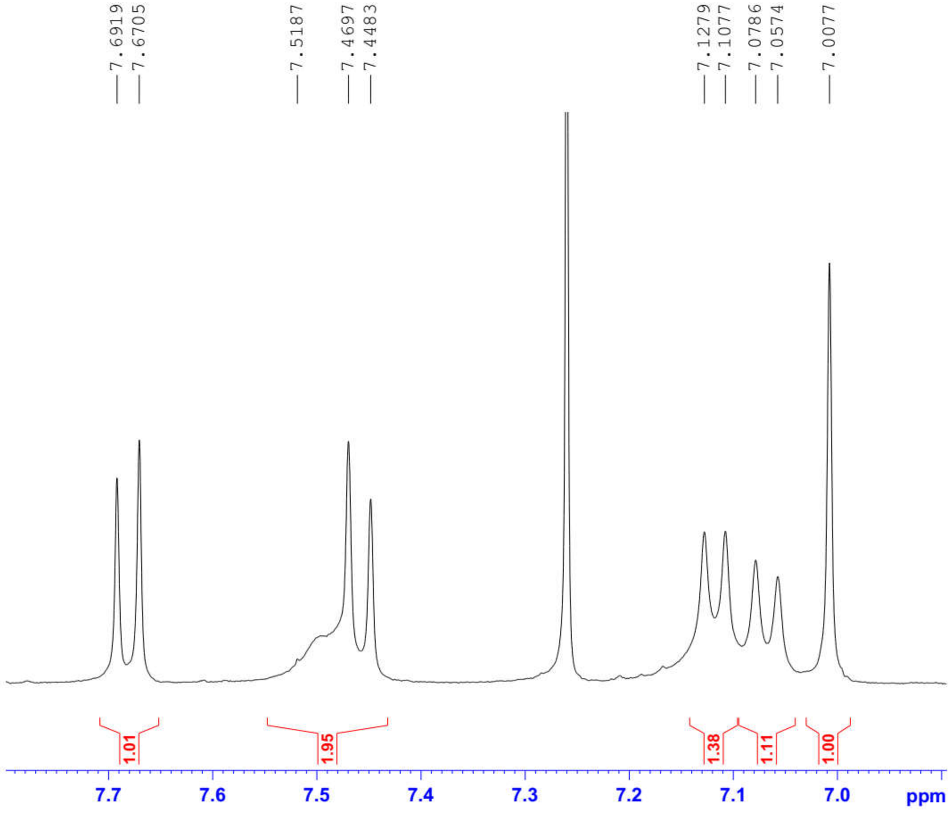

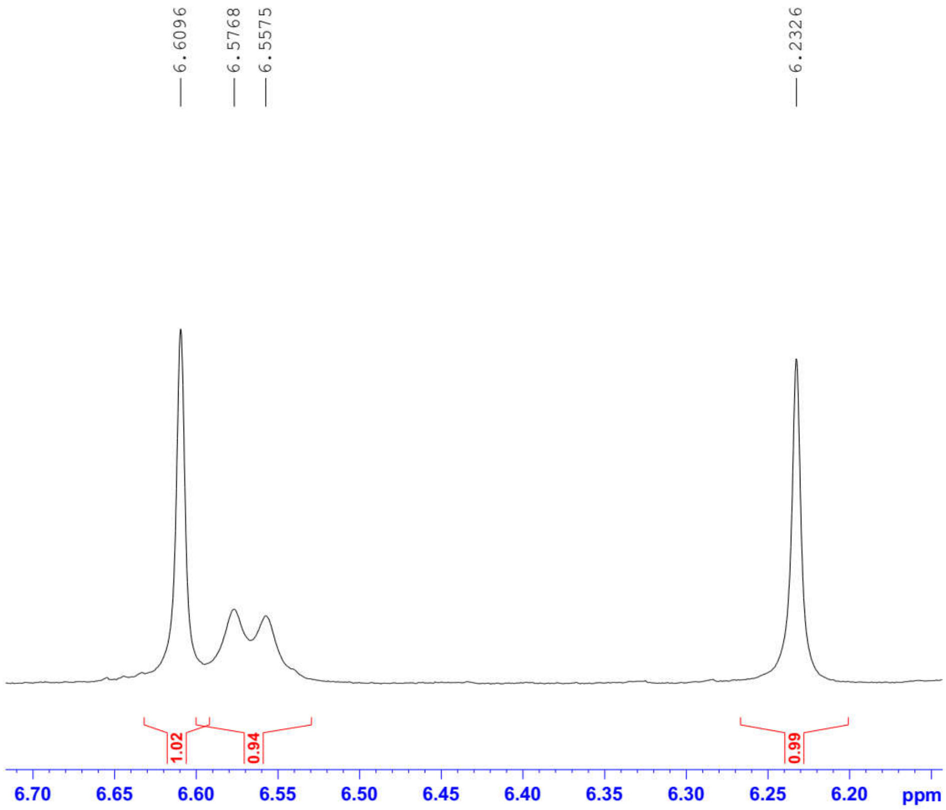
6.6096
6.5768
6.5575
6.2326
1.02
0.94
0.99
6.70 6.65 6.60 6.55 6.50 6.45 6.40 6.35 6.30 6.25 6.20 ppm

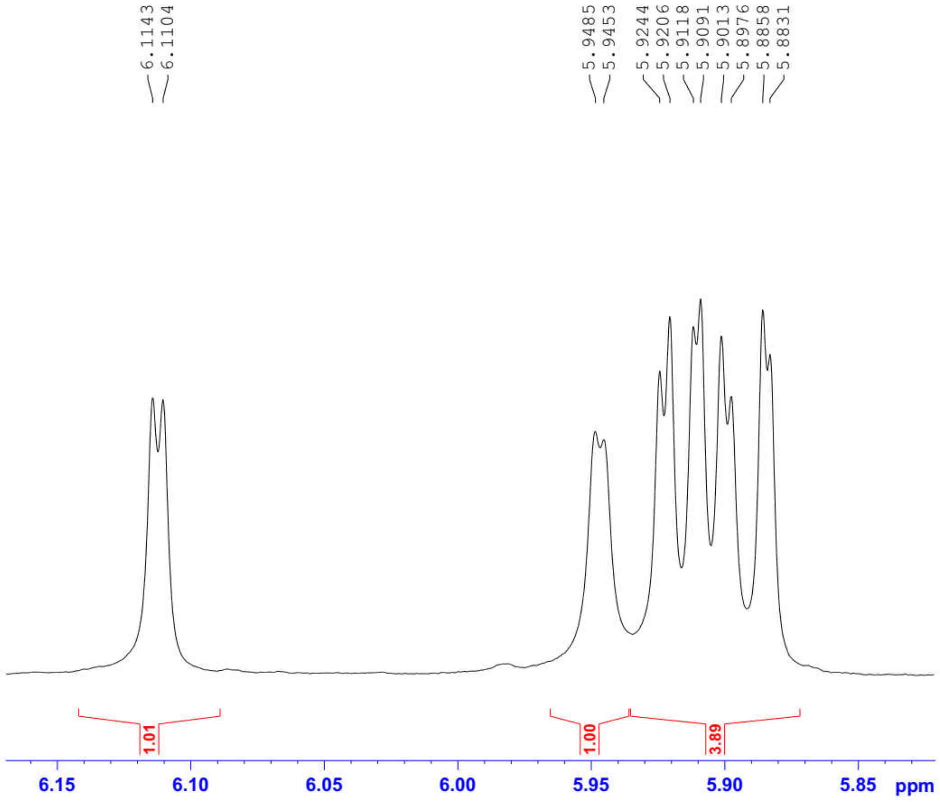
6.1143
6.1104
5.9485
5.9453
5.9244
5.9206
5.9118
5.9091
5.9013
5.8976
5.8858
5.8831
1.01
1.00
3.89
6.15 6.10 6.05 6.00 5.95 5.90 5.85 ppm

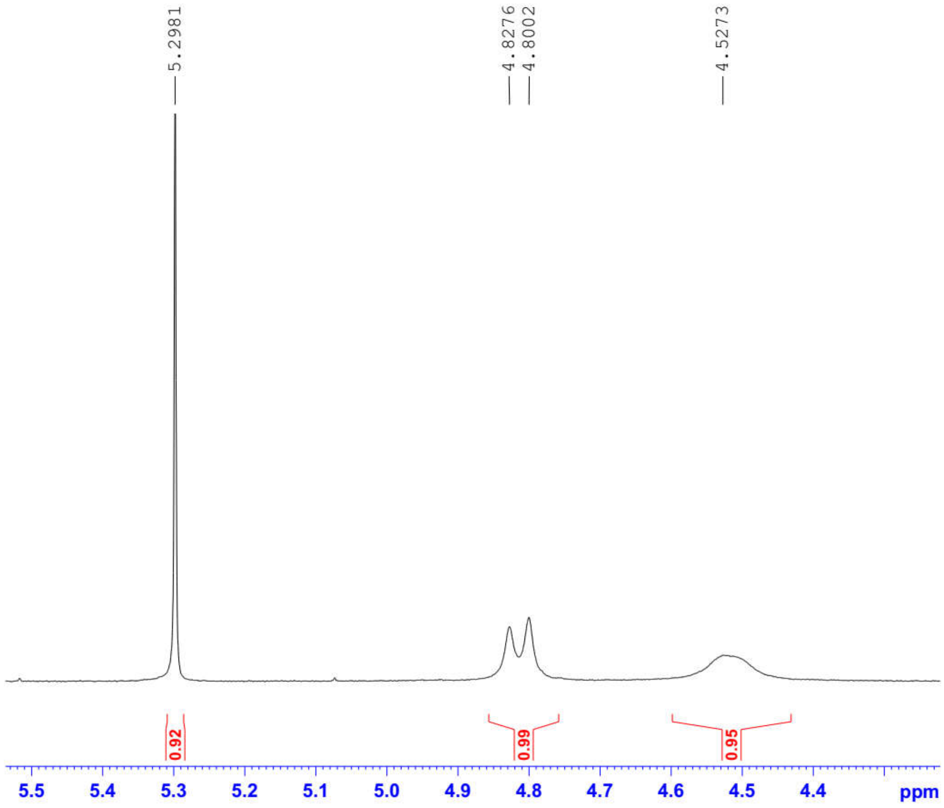
5.2981
4.8276
4.8002
4.5273
0.92
0.99
0.95
5.5
5.4
5.3
5.2
5.1
5.0
4.9
4.8
4.7
4.6
4.5
4.4
ppm

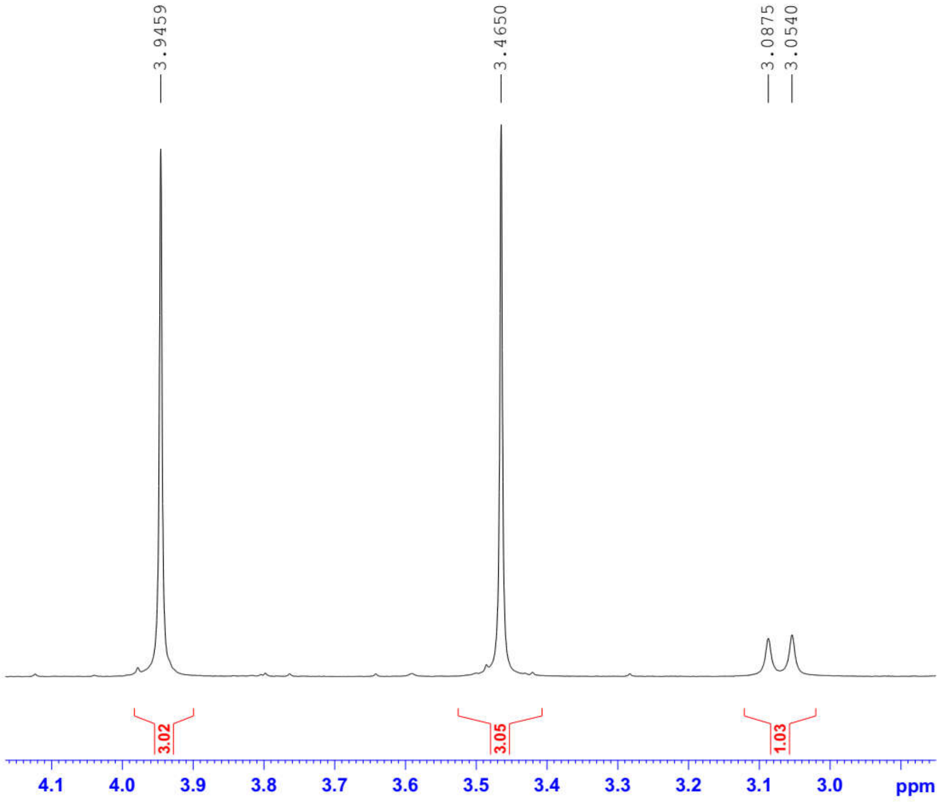
3.9459
3.4650
3.0875
3.0540
3.02
3.05
1.03
4.1
4.0
3.9
3.8
3.7
3.6
3.5
3.4
3.3
3.2
3.1
3.0
ppm

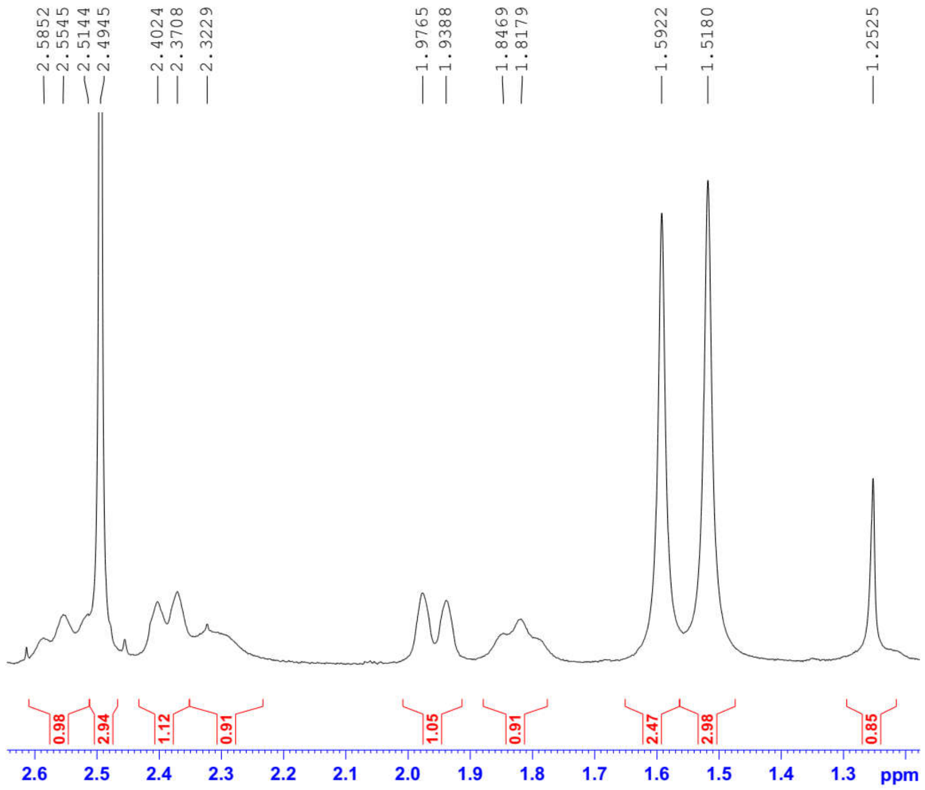

2 Data of in vitro cytotoxicity assay and determination by CCK-8

Hela 72 h Compound: Macleayin A IC_{50} = 26.878 μM

**Group（conc.）**	control	0.39μM	0.78μM	1.56μM	3.125μM	6.25μM	12.5μM	25μM	50μM	100μM
**log（conc.）**		-0.41	-0.11	0.19	0.49	0.80	1.10	1.40	1.70	2.00
	1.621	1.6075	1.574	1.5427	1.3266	1.2437	1.0471	0.8283	0.5242	0.2148
**OD**	1.594	1.5826	1.5202	1.472	1.4598	1.1995	1.056	0.914	0.5638	0.1837
	1.612	1.5931	1.5083	1.5357	1.3905	1.2665	1.0674	0.9508	0.6005	0.1264
		0.81	2.88	4.81	18.14	23.26	35.39	48.89	67.65	86.75
**inhibition ratio**		0.72	4.64	7.66	8.42	24.75	33.76	42.66	64.63	88.48
		1.18	6.43	4.73	13.74	21.43	33.78	41.02	62.75	92.16
**inhibition ratio Mean%**		0.90	4.65	5.73	13.44	23.15	34.31	44.19	65.01	89.13
**SD**		0.25	1.78	1.67	4.87	1.66	0.93	4.15	2.47	2.76

HepG2 72 h Compound：Macleayin A

group（conc.）	control	0.39μM	0.78μM	1.56μM	3.125μM	6.25μM	12.5μM	25μM	50μM	100μM
log（conc.）		-0.41	-0.11	0.19	0.49	0.80	1.10	1.40	1.70	2.00
	2.4157	2.3543	2.3164	2.2729	2.1410	2.1601	2.0163	2.0655	1.8946	1.8016
OD	2.4601	2.3564	2.3155	2.1667	2.1213	2.0964	2.0064	1.9297	1.9418	1.8823
	2.4441	2.2381	2.2839	2.1362	2.2055	2.0468	2.1094	1.9557	1.9180	1.7870
		2.54	4.11	5.91	11.37	10.58	16.53	14.50	21.57	25.42
inhibition ratio		4.22	5.88	11.93	13.77	14.78	18.44	21.56	21.07	23.49
		8.43	6.55	12.60	9.76	16.26	13.69	19.98	21.53	26.89
inhibition ratio mean%		5.06	5.51	10.15	11.64	13.87	16.22	18.68	21.39	25.26
SD		3.10	1.28	3.75	2.06	3.00	2.45	3.78	0.30	1.76

siHa 72h Compound：Macleayin A IC50=667.249 μM

group（conc.）	control	0.39μM	0.78μM	1.56μM	3.125μM	6.25μM	12.5μM	25μM	50μM	100μM
log（conc.）		-0.41	-0.11	0.19	0.49	0.80	1.10	1.40	1.70	2.00
	1.7353	1.6392	1.6080	1.6093	1.5831	1.5428	1.5685	1.5010	1.4173	1.3652
OD	1.6377	1.7470	1.6562	1.6836	1.6027	1.5666	1.5154	1.4762	1.3012	1.2204
	1.6578	1.6365	1.6969	1.6214	1.6637	1.5854	1.5296	1.5075	1.4123	1.3335
		5.5380	7.3359	7.2610	8.7708	11.0931	9.6122	13.502	18.325	21.328
inhibition ratio		-6.674	-1.1296	-2.8027	2.1371	4.3414	7.4678	9.8614	20.547	25.481
		1.2848	-2.3585	2.1957	-0.3559	4.3672	7.7331	9.0662	14.809	19.562
inhibition ratio mean%		0.16	1.39	2.32	3.60	6.68	8.29	10.86	17.89	22.10
SD	6.20	5.28	5.03	4.72	3.89	1.17	2.37	2.89	3.04	6.20

HFL-1 72 h Compound：Macleayin A IC50=485353.837 μM

group（conc.）	control	0.39μM	0.78μM	1.56μM	3.125μM	6.25μM	12.5μM	25μM	50μM	100μM
log（conc.）		-0.41	-0.11	0.19	0.49	0.80	1.10	1.40	1.70	2.00
	1.1743	1.1792	1.1102	1.1261	1.1383	1.0947	1.0797	1.1508	1.0953	1.0731
OD	1.1414	1.1336	1.0859	1.1227	1.1053	1.1652	1.0693	1.0283	1.0981	1.0381
	1.2852	1.2351	1.2342	1.1312	1.1221	1.0875	1.1492	1.0705	1.0558	1.0882
		-0.42	5.46	4.10	3.07	6.78	8.06	2.00	6.73	8.62
inhibition ratio		0.68	4.86	1.64	3.16	-2.09	6.32	9.91	3.79	9.05
		3.90	3.97	11.98	12.69	15.38	10.58	16.71	17.85	15.33
inhibition ratio mean%		1.39	4.76	5.91	6.31	6.69	8.32	9.54	9.46	11.00
SD		2.24	0.75	5.40	5.53	8.73	2.14	7.36	7.41	3.76

3 Data of cell proliferation assay by EdU labeling method

Group	Proliferative cell	Total cell	Proliferation rate	Mean proliferation rate
control（3）	45	84	53.57%	
control（6）	50	98	51.02%	
control（9）	47	85	55.29%	
control（12）	49	106	46.23%	
control（15）	50	100	50.00%	52.46%
control（18）	55	95	57.89%	
control（21）	59	109	54.13%	
control（24）	48	99	48.48%	
control（27）	40	72	55.56%	
6.75 **μM**（3）	39	89	43.82%	
6.75 **μM**（6）	37	80	46.25%	
6.75 **μM**（9）	30	75	40.00%	
6.75 **μM**（12）	41	95	43.16%	
6.75 **μM**（15）	35	82	42.68%	43.85%
6.75 **μM**（18）	37	92	40.22%	
6.75 **μM**（21）	30	65	46.15%	
6.75 **μM**（24）	28	68	41.18%	
6.75 **μM**（27）	42	82	51.22%	
13.5 **μM**（3）	30	82	36.59%	
13.5 **μM**（6）	28	83	33.73%	
13.5 **μM**（9）	24	71	33.80%	
13.5 **μM**（12）	27	84	32.14%	
13.5 **μM**（15）	25	86	29.07%	34.71%
13.5 **μM**（18）	30	88	34.09%	
13.5 **μM**（21）	34	89	38.20%	
13.5 **μM**（24）	26	78	33.33%	
13.5 **μM**（27）	16	69	23.19%	
27.0 **μM**（3）	13	64	20.31%	
27.0 **μM**（6）	15	67	22.39%	
27.0 **μM**（9）	20	80	25.00%	
27.0 **μM**（12）	16	74	21.62%	
27.0 **μM**（15）	14	69	20.29%	22.57%
27.0 **μM**（18）	19	80	23.75%	
27.0 **μM**（21）	14	68	20.59%	
27.0 **μM**（24）	16	71	22.54%	
27.0 **μM**（27）	22	75	29.33%	

4 Data of cell apoptosis assay by Annexin-V APC/7-AAD double staining method

Group	UL(%)	UR(%)	LL(%)	LR(%)	Apoptosis(%)	Mean apoptosis%	SD
**control-1**	0.39	1.54	93.55	4.53	6.07		
**control-2**	0.66	2.09	91.85	5.4	7.49	7.00	0.80
**control-3**	0.46	1.52	92.11	5.91	7.43		
6.75 **μM -1**	1.64	11.23	74.13	13	24.23		
6.75 **μM -2**	1.33	11.14	74.93	12.6	23.74	23.93	0.26
6.75 **μM -3**	1.87	11.63	74.3	12.2	23.83		
13.5 **μM -1**	5	17.59	59.39	18.02	35.61		
13.5 **μM -2**	4.29	15.56	60.16	19.99	35.55	35.45	0.23
13.5 **μM -3**	5.59	16.24	59.21	18.95	35.19		
27.0 **μM -1**	1.76	14.68	51.76	31.81	46.49		
27.0 **μM -2**	1.92	12.95	53.91	31.22	44.17	43.93	2.69
27.0 **μM-3**	1.32	11.34	57.55	29.79	41.13		

5 Data of cell cycle assay by PI staining method

Group	G1(%)	S(%)	G2(%)
control-1	47.58	46.41	6.01
control-2	51.03	43.47	5.50
control-3	58.54	35.39	6.07
control-Mean	52.38	41.76	5.86
control-SD	5.60	5.71	0.31
6.75 **μM** -1	68.27	27.64	4.09
6.75 **μM** -2	67.53	27.98	4.50
6.75 **μM** -3	69.96	25.27	4.77
6.75 **μM** -Mean	68.59	26.96	4.45
6.75 **μM** -SD	1.25	1.48	0.34
13.5 **μM** -1	72.79	23.71	3.50
13.5 **μM** -2	74.17	22.62	3.21
13.5 **μM** -3	75.06	22.11	2.83
13.5 **μM** -Mean	74.01	22.81	3.18
13.5 **μM** -SD	1.14	0.82	0.34
27.0 **μM** -1	72.11	24.62	3.27
27.0 **μM** -2	73.77	24.21	2.01
27.0 **μM** -3	77.72	20.85	1.43
27.0 **μM** -Mean	74.53	23.23	2.24
27.0 **μM** -SD	2.88	2.07	0.94

6 Data of cell migration and invasion assay by transwell

Group	Migration Cell Number										
	1	2	3	4	5	6	7	8	9	Mean	SD
**Control**	320	325	317	321	322	324	325	323	327	322.67	3.04
**6.75 μM**	298	291	295	286	287	292	290	284	288	290.11	4.46
**13.5 μM**	215	216	210	208	214	212	213	215	219	213.56	3.28
**27.0 μM**	135	140	143	145	146	148	143	142	134	141.78	4.74

Group	Invasion Cell Number										
	1	2	3	4	5	6	7	8	9	Mean	SD
**Control**	243	238	236	237	241	249	248	236	240	240.89	4.91
**6.75 μM**	178	171	175	176	167	174	179	173	182	175.00	4.47
**13.5 μM**	85	86	83	90	87	85	83	82	86	85.22	2.44
**27.0 μM**	57	65	63	52	62	59	64	60	64	60.67	4.18

7 Data of protein expression assay by Western blot

	Control	6.75 μM	13.5 μM	27.0 μM	Control	6.75 μM	13.5 μM	27.0 μM	Control	6.75 μM	13.5 μM	27.0 μM
**β-catenin**	370.15	216.19	137.06	90.852	299.47	228.47	116.6	84.981	269.86	176.02	100.53	70.679
**MMP-7**	463.05	242.39	104.62	77.939	527.22	274.25	127.79	51.854	419.99	210.03	152.04	111.75
**cyclin D1**	700.12	453.17	294.17	162.22	768	454.03	243.59	150.93	469.12	403.8	232.03	131.7
**c-myc**	400.56	237.52	190.84	108.81	351.65	204.51	129.89	94.345	321.55	181.54	109.67	57.707
**GAPDH**	1372.3	1475.5	1522	1627.8	1514.1	1467.7	1547.5	1486.5	1658.7	1535.9	1419.2	1252.6

	Control	6.75 μM	13.5 μM	27.0 μM	Control	6.75 μM	13.5 μM	27.0 μM	Control	6.75 μM	13.5 μM	27.0 μM
**β-catenin/GAPDH**	0.270	0.147	0.090	0.056	0.198	0.156	0.075	0.057	0.163	0.115	0.071	0.056
**MMP-7/GAPDH**	0.337	0.164	0.069	0.048	0.348	0.187	0.083	0.035	0.253	0.137	0.107	0.089
**cyclin D1/GAPDH**	0.510	0.307	0.193	0.100	0.507	0.309	0.157	0.102	0.283	0.263	0.163	0.105
**c-myc/GAPDH**	0.292	0.161	0.125	0.067	0.232	0.139	0.084	0.063	0.194	0.118	0.077	0.046
**β-catenin/GAPDH**	0.270	0.147	0.090	0.056	0.198	0.156	0.075	0.057	0.163	0.115	0.071	0.056

8 Data of Luciferase reporter assay

	control	6.75 μM	13.5 μM	27.0 μM
	1336.458	1026.315	816.315	623.523
**Luc fluorescence intensity**	1289.374	1056.301	817.27	595.721
	1221.023	988.415	735.54	582.299
	205.354	196.247	189.342	145.24
**Rluc fluorescence intensity**	199.689	182.354	193.54	158.36
	187.047	177.364	189.374	152.314
	6.51	5.23	4.31	4.29
**Luc/Rluc**	6.46	5.79	4.22	3.76
	6.53	5.57	3.88	3.82
**mean**	6.50	5.53	4.14	3.96
**SD**	0.04	0.28	0.23	0.29
